# The Efficacy and Safety of Doripenem in the Treatment of Acute Bacterial Infections—A Systemic Review and Meta-Analysis of Randomized Controlled Trials

**DOI:** 10.3390/jcm8070958

**Published:** 2019-07-02

**Authors:** Chih-Cheng Lai, I-Ling Cheng, Yu-Hung Chen, Hung-Jen Tang

**Affiliations:** 1 Department of Intensive Care Medicine, Chi Mei Medical Center, Liouying 73657, Taiwan; 2 Department of Pharmacy, Chi Mei Medical Center, Liouying 73657, Taiwan; 3 Department of Medicine, Chi Mei Medical Center, Tainan 71004, Taiwan

**Keywords:** doripenem, acute bacterial infection, pneumonia, intra-abdominal infection, complicated urinary tract infection

## Abstract

This study aims to assess the efficacy and safety of doripenem on treating patients with acute bacterial infections. The Pubmed, Embase, and Cochrane databases were searched up to April 2019. Only randomized clinical trials comparing doripenem and other comparators for the treatment of acute bacterial infection were included. The primary outcome was the clinical success rate and the secondary outcomes were microbiological eradication rate and risk of adverse events. Eight randomized controlled trials (RCTs) were included. Overall, doripenem had a similar clinical success rate with comparators (odds ratio [OR], 1.15; 95% CI, 0.79–1.66, I2 = 58%). Similar clinical success rates were noted between doripenem and comparators for pneumonia (OR, 0.84; 95% CI, 0.46–1.53, *I*^2^ = 72%) and for intra-abdominal infections (OR, 1.00; 95% CI, 0.57–1.72). For complicated urinary tract infection, doripenem was associated with higher success rate than comparators (OR, 1.89, 95% CI, 1.13–3.17, *I*^2^ = 0%). The pool analysis comparing doripenem and other carbapenems showed no significant differences between each other (OR, 0.96, 95% CI, 0.59–1.58, *I*^2^ = 63%). Doripenem also had a similar microbiological eradication rate with comparators (OR, 1.08; 95% CI, 0.86–1.36, *I*^2^ = 0%). Finally, doripenem had a similar risk of treatment-emergent adverse events as comparators (OR, 0.98; 95% CI, 0.83–1.17, *I*^2^ = 33%). In conclusion, the clinical efficacy of doripenem is as high as that of the comparator drugs in the treatment of acute bacterial infection; furthermore, this antibiotic is as well tolerated as the comparators.

## 1. Introduction

Carbapenems, including imipenem and meropenem, remain the mainstay of treatment for hospital-acquired infections, especially for the multidrug-resistant organism associated infections [1]. Doripenem is another important carbapenem, and has excellent bactericidal activity against most nosocomial pathogens according to several in vitro studies [2,3,4,5]. A global surveillance showed that doripenem was at least two-fold more potent in in vitro activities than imipenem and meropenem against *Pseudomonas aeruginosa*—an important nosocomial pathogen [3]. For another notorious pathogen—*Acinetobacter baumannii*, doripenem displayed comparable in vitro activities to imipenem and meropenem [4]. Clinically, doripenem is approved for the treatment of patients with complicated intra-abdominal infection (cIAI), complicated urinary tract infection (cUTI) and pyelonephritis, and healthcare-associated pneumonia (HAP) including ventilator-associated pneumonia (VAP) in Europe and in other countries, other than United States. Although Qu et al. [6] conducted a meta-analysis of doripenem for treating bacterial infections in 2015, only six clinical trials were enrolled and the number of patients was limited. Since then, two more studies investigating the efficacy of doripenem in comparison with other comparators were reported [7,8]. In Wagenlehner et al.’s study [7], 1033 randomized patients were enrolled, and they did the comparison between doripenem and ceftazidime-avibactam for the treatment of cUTI. In Oyake et al.’s study [8], they compared the empirical use of doripenem versus meropenem for febrile neutropenia in patients with acute leukemia. These two studies provided more patients and different types of infections compared to previse meta-analysis [6]. Therefore, we could conduct a comprehensive review and updated meta-analysis to assess the efficacy and safety of doripenem on treating patients with acute bacterial infections in comparison with other antibiotics, especially imipenem and meropenem.

## 2. Methods

### 2.1. Study Search and Selection

Studies were identified by a systematic review of the literature in the PubMed, Embase, and Cochrane databases until April 2019 using the following search terms—“doripenem,” “infection,” and “randomized” (Appendix A). Studies were considered eligible for inclusion if they directly compared the clinical effectiveness of doripenem with other antimicrobial agents in the treatment of adult patients with acute bacterial infections. Studies were excluded if they focused on in vitro activity, or pharmacokinetic-pharmacodynamic assessment. The articles of all languages of publication could be included. Two reviewers (I.-L.C. and Y.-H.C.) searched and examined publications independently. When they had disagreement, the third author (C.-C.L.) resolved the issue in time. The following data including year of publication, study design, type of infections, patients’ demographic features, antimicrobial regimens, clinical and microbiological outcomes, and adverse events were extracted from every included study.

### 2.2. Definitions and Outcomes 

The primary outcome was overall clinical success with resolution of clinical signs and symptoms of acute bacterial infection, or recovery to the pretreatment state at the end of treatment. Secondary outcomes included the microbiological eradication rate and adverse events. A microbiological eradication was defined as eradication (the baseline pathogen was absent) and presumed eradication (if an adequate source specimen was not available to culture, but the patient was assessed as clinically cured). Treatment-emergent adverse events were recorded, irrespective of causality. In addition, the risk of discontinuing due to adverse event and the incidence of serious adverse events, and some common events, including diarrhea, nausea, headache, constipation, and seizure were recorded.

### 2.3. Data Analysis

This study used the Cochrane risk of bias assessment tool to assess the quality of enrolled randomized controlled trials (RCTs) and the risk of bias [9]. The software Review Manager, version 5.3, was used to conduct the statistical analyses. The degree of heterogeneity was evaluated with the Q statistic generated from the χ^2^ test. The proportion of statistical heterogeneity was assessed by the *I^2^* measure. Heterogeneity was considered significant when the p-value was less than 0.10 or the *I^2^* was more than 50%. The random-effects model was used when the data were significantly heterogeneous, and the fixed-effect model was used when the data were homogenous. Pooled odds ratios (OR) and 95% confidence intervals (CI) were calculated for outcome analyses. Sensitivity analysis was performed to ensure that the findings were not significantly affected by any individual study

## 3. Results

### 3.1. Study Selection and Characteristics

**Figure 1:**
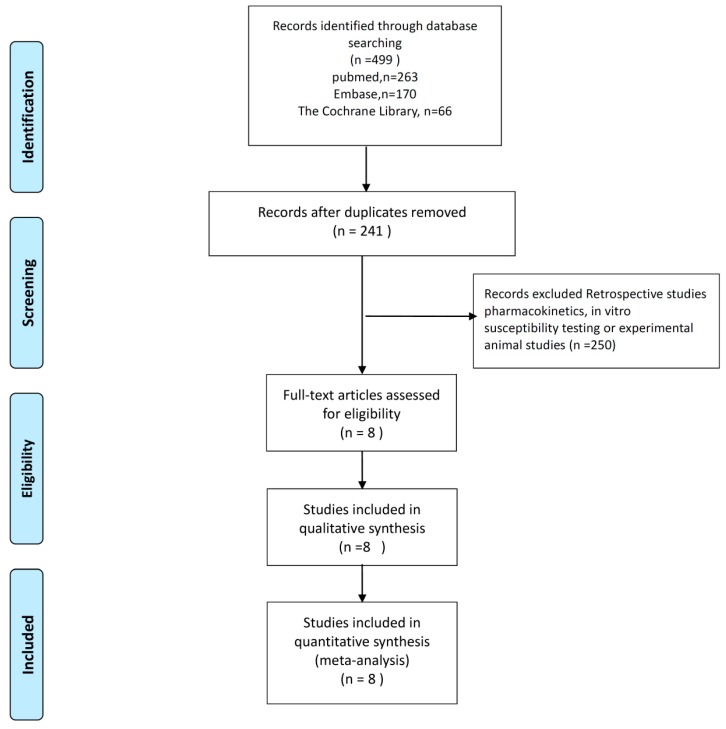
Flowchart of the study selection process.

**Figure 2:**
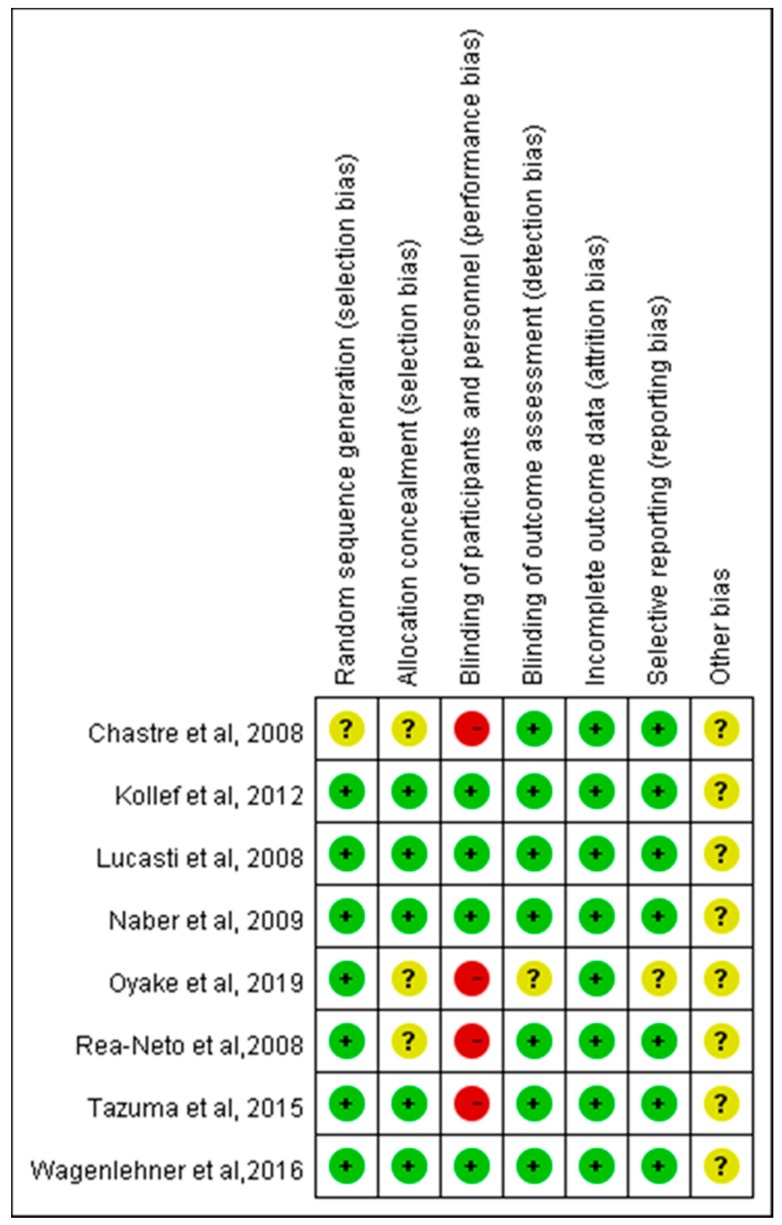
Risk of bias per study and domain.

The search program yielded 499 references, including 263 from Pubmed, 170 from Embase, and 66 from Cochrane database. Then, 258 articles were screened after excluding 241 duplicated articles. Finally, a total of eight RCTs [7,8,10,11,12,13,14,15] fulfilling the inclusion criteria were included in this meta-analysis (Figure 1). All of studies were designed to compare the clinical efficacy and safety of doripenem with other antibiotics for patients with acute bacterial infection (Table 1) [7,8,10,11,12,13,14,15]. During the initial enrollment, doripenem and comparators were applied to 1736 and 1763 patients, respectively. Six studies [7,10,11,12,13,15] of them were multicenter studies. Three studies [10,11,12] focused on pneumonia, including two [12,16] on ventilator-associated pneumonia and one [10] on nosocomial pneumonia. Two studies focused on complicated urinary tract infections (cUTI) [7,13] and intra-abdominal infections (IAI) [14,15]. Only one study investigated febrile neutropenia [8]. Five studies [8,11,12,14,15] compared doripenem with other carbapenems including imipenem in three studies [11,12,14] and meropenem in two studies [8,15]. The regimen of doripenem was 1 g every eight hours in two studies [8,11] and 500 mg every eight hours in the other six studies [7,10,12,13,14,15]. For the two studies using double dose of doripenem (1 g every eight hour), the study drug (doripenem or meropenem) was used for at least five days in one study [8] and another one [11] compared seven-day doripenem versus 10-day imipenem-cilastatin. Figure 2 shows the analyses of risk of bias.

### 3.2. Clinical Success

**Figure 3:**
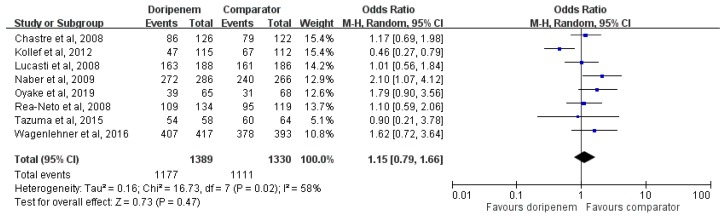
Overall clinical success rates of doripenem and comparators in the treatment of acute bacterial infections.

Overall, doripenem had a similar clinical success rate with comparators (OR, 1.15; 95% CI, 0.79–1.66, *I^2^* = 58%, Figure 3). Sensitivity analysis after randomly deleting an individual study each time to reflect the influence of the single data set to the pooled OR showed similar findings in most occasions. There was only one exception, when we deleted Kollef et al.’s study [11], doripenem showed better clinical success rate than other comparators in the pool analysis of the remaining seven studies [7,8,10,12,13,14,15] (OR, 1.33; 95% CI, 1.03–1.72, *I^2^* = 0%). In the different subgroup of patients with pneumonia, cUTI, and intra-abdominal infection, similar clinical success rates were noted between two different regimens for pneumonia (OR, 0.84; 95% CI, 0.46–1.53, *I^2^* = 72%) and for IAI (OR, 1.00; 95% CI, 0.57–1.72). For cUTI, doripenem was associated with a higher success rate than comparators (OR, 1.89, 95% CI, 1.13–3.17, *I^2^* = 0%). Three studies [11,12,14] compared the effect of doripenem and imipenem, and there was no difference in terms of clinical success rate between these two regimens (OR, 0.76; 95% CI, 0.38–1.55, *I^2^* = 66%). Two studies [8,15] compared doripenem and meropenem, their clinical success rates were similar (OR, 1.31, 95% CI, 0.75–2.28, *I^2^* = 34%). The pool analysis of these five studies comparing doripenem and other carbapenems showed no significant differences between each other (OR, 0.96, 95% CI, 0.59–1.58, *I^2^* = 63%).

### 3.3. Microbiological Eradication

**Figure 4:**
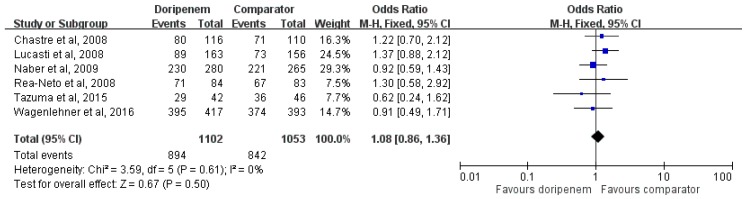
Overall microbiological eradication rates of doripenem and comparators in the treatment of acute bacterial infections.

Only six studies [7,10,12,13,14,15] reported the data of microbiological eradication rate, and the pool analysis showed that doripenem had a similar microbiological eradication rate with comparators (OR, 1.08; 95% CI, 0.86–1.36, *I^2^* = 0%, Figure 4). Sensitivity analysis showed similar results. In the different subgroup of patients with pneumonia and IAI, similar microbiological eradication rates were found for both regimens (for pneumonia, OR, 1.25; 95% CI, 0.79–1.97, *I^2^* = 0%; for IAI, OR, 1.04; 95% CI, 0.49–2.17, *I^2^* = 54%). While comparing doripenem and other carbapenems in the pool analysis of four studies [7,12,14,15], the microbiological eradication rates were similar between these two regimens (OR, 1.13; 95% CI, 0.85–1.51, *I^2^* = 0%).

### 3.4. Adverse Events

**Figure 5:**
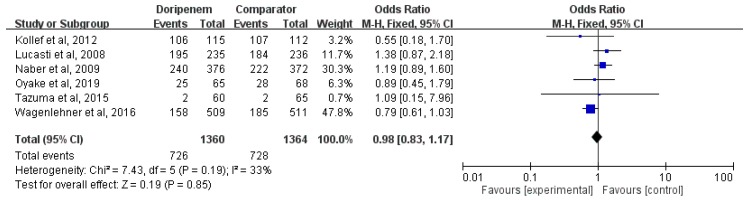
Risk of treatment-emergent adverse events of doripenem and comparators in the treatment of acute bacterial infections.

Six studies [7,8,11,13,14,15] reported the incidence of treatment-emergent adverse events, the doripenem had a similar risk with other antibiotics (OR, 0.98; 95% CI, 0.83–1.17, *I^2^* = 33%, Figure 5). Serious adverse events were reported in six studies [7,10,12,13,14,15], the overall incidence was similar between doripenem and other antibiotics (OR, 1.06; 95% CI, 0.85–1.31, *I*^2^ = 43%). Six studies [7,10,12,13,14,15] reported the risk of discontinuing drug due to adverse event, the risk was similar between doripenem and comparators (OR, 0.75, 95% CI, 0.35–1.61, *I^2^* = 61%). Regarding some common adverse events, doripenem was associated with the similar risk as comparators in terms of diarrhea (OR, 0.91, 95% CI, 0.64–1.28, *I^2^* = 0%) in the pool analysis of eight studies [7,8,10,11,12,13,14,15], nausea (OR, 0.93, 95% CI, 0.45–1.93, *I^2^* = 62%) among five studies [11,12,13,14,15], headache (OR, 1.10, 95% CI, 0.82–1.48, *I^2^* = 0%) among three studies [13,14,15], and constipation (OR, 0.96, 95% CI, 0.61–1.52, *I^2^* = 0%) among three studies [11,13,14]. In the pooled analysis of four studies [10,12,13,15] that reported the risk of seizure, doripenem was associated with a similar lower risk as comparators (OR, 0.37, 95% CI, 0.15–0.92, *I^2^* = 0%). Moreover, no seizure attack was reported to be related to doripenem in these four studies [10,12,13,15].

## 4. Discussion

This meta-analysis based on eight RCTs found that doripenem had a similar clinical success rate of treating acute bacterial infections with other comparators. The similar efficacy in terms of clinical response and microbiological eradication was found between doripenem and other carbapenems, including meropenem and imipenem. In addition, this result was not affected by the different types of infections—pneumonia, cUTI, or IAIs. Even for several specific types of infection—cholangitis, cholecystitis, appendicitis, lower urinary tract infection, and acute pyelonephritis—no statistical differences in terms of clinical efficacy was found between doripenem and comparators in the included studies [13,14,15]. In fact, in addition to Kollef et al.’s study [11], that showed doripenem was found to have non-significant higher rates of clinical failure and mortality compared to imipenem [7,10,12,13,14,15]. The difference between Kollef et al.’s study [11] and the other seven studies [7,10,12,13,14,15] may be explained by the fact that Kollef et al. compared a fixed seven-day course of doripenem with a fixed 10-day course of imipenem-cilastatin for treating VAP. Seven days of antibiotic treatment may have been too short for the patients with VAP, so the clinical outcome of VAP treated with a seven-day course of doripenem was worse than with a 10-day course of imipenem-cilastatin. In this meta-analysis, while we did sensitivity analysis after deleting this negative study [11] for doripenem, we found that the pooled analysis of the other seven studies [7,8,10,12,13,14,15] showed that doripenem was associated with better clinical outcome than comparators. Although this finding hints that the effect of doripenem may be better, or at least as good as, other antimicrobial agents in the treatment of acute bacterial infections, if doripenem can be used as long as the comparators, we still need further study to confirm this issue. Before that, the findings of this meta-analysis indicate that the clinical efficacy of doripenem is not inferior to other antimicrobial agents in the treatment of acute bacterial infections. Finally, several studies [16,17,18] demonstrated that doripenem was associated with lower medical resource utilization and hospital cost in the treatment of HAP and VAP versus comparators, including imipenem. Overall, doripenem could be both a life- and cost-saving antibiotic and could be recommended as the appropriate antibiotic in the treatment of acute bacterial infections, including pneumonia, cUTI, and cIAI.

In this meta-analysis, we also compared the microbiological response of doripenem with other antibiotics for acute bacterial infection. Overall, we found the microbiological eradication rates were similar between doripenem and comparators. Moreover, a similar trend was noted in the sensitivity analysis and subgroup analysis of pneumonia and IAIs. Finally, doripenem was comparable to other carbapenems, including imipenem and meropenem, in terms of microbiological eradication rate in the subgroup analysis. All these findings may be well explained by previous in vitro studies [3,4,19,20,21] that showed doripenem had a greater or similar in vitro activity against bacteria, including multi-drug resistant organisms. In this meta-analysis, we did not assess we did not evaluate the association between in vitro activity and the in vivo response of different organisms, especially for antibiotic-resistant pathogens, because the associated information was limited. However, this meta-analysis demonstrates that doripenem is comparable to other antimicrobial agents in both the clinical and microbiological responses of treating acute bacterial infections.

In addition to the assessment of clinical efficacy and microbiological eradication, the safety issue is another important concern in the treatment of acute bacterial infection by doripenem. In this analysis, the risks of overall treatment-emergent adverse effects, common adverse effects (diarrhea, nausea, headache and constipation), serious adverse effects, and the risk of discontinuing the drug due to adverse effects were similar between doripenem and comparators. Seizure is another important concern for patients using carbapenems. In this meta-analysis, four studies [10,12,13,15] reported the incidence of seizure, and the doripenem group had a lower risk of seizure than comparators. Moreover, although six seizure events were reported in this meta-analysis, all these events occurred in patients with underlying risk factors and were not clearly related to doripenem. Therefore, all these findings indicate that doripenem may be as safe as conventional regimens in the treatment of acute bacterial infections.

This meta-analysis has several limitations. First, we did not evaluate the effect of doripenem and comparators against specific organisms in each type of bacterial infection and the confounding effect of the antibiotic resistance of these pathogens. Besides, the immune status and the age effect were not assessed in this meta-analysis due to limited information. Second, the use of doripenem for treating pneumonia remains a serious concern due to the negative findings of Kolleff et al.’s study [11] that showed a shorter course (seven days) of doripenem was associated with a worse outcome than a longer course (10 days) of imipenem for patients with VAP. However, doripenem was commonly used for treating pneumonia in many countries [22], and several studies [10,12,23,24,25] showed the clinical outcomes of pneumonia treated by doripenem were favorable. In the subgroup analysis of this meta-analysis, we found the clinical and microbiological responses of doripenem for treating pneumonia were as good as comparators. But, as only three RCTs [10,11,12] focusing on pneumonia were enrolled in this meta-analysis, the number of studies is limited, thus further study is warranted to clarify this issue.

In conclusion, based on the analysis of eight RCTs, no differences in terms of clinical success and microbiological eradication rates were found between doripenem and comparators in the treatment of acute bacterial infections. Moreover, doripenem was well tolerated and had comparable safety profiles to other antimicrobial agents.

## Figures and Tables

**Table 1:** Characteristics of included studies.

Reference	RCT Study Design	Duration	Study Population	No. of Patients		Age of the Patients		Dose Regimen	
				Doripenem	Comparator	Doripenem	Comparator	Doripenem	Comparator
[10]	Randomized, open-label, multicenter trial	2004–2006	Nosocomial pneumonia	225	223	57.5	59.3	Doripenem 500 mg every 8 h	Piperacillin/tazobactam 4.5 g every 6 h
[15]	Prospective, multicenter, randomized, double-blind	2004–2006	Complicated intra-abdominal infection	237	239	46.9	46.4	Doripenem 500 mg every 8 h	Meropenem 1.0 g every 8 h
[12]	Prospective, multicenter, randomized, open-label trial	2004–2006	Ventilator-associated pneumonia	262	263	50.7	50.3	Doripenem 500 mg every 8 h	Imipenem/cilastatin 1 g every 8 h or 500 mg every 6 h
[13]	Prospective, multicenter, double-blind trial	2003–2006	Complicated UTI	377	376	51.2	51.1	Doripenem 500 mg every 8 h	Levofloxacin 250 mg everyday
[11]	Randomized, double-blind, multicenter trial	2008–2011	Ventilator-associated pneumonia	115	112	57.5	54.6	Doripenem 1 g every 8 h	Imipenem/cilastatin 1 g every 8 h
[14]	Randomized, open-label trial	2010–2013	Moderate or severe acute cholangitis or cholecystitis	62	65	74	73	Doripenem 500 mg every 8 h	Imipenem/cilastatin 500 mg every 8 h
[7]	Randomized, multicenter, double-blind, trials	2012–2014	Complicated UTI	393	417	53.3	51.4	Doripenem 500 mg every 8 h	Ceftazidime-avibactam 2000 mg/500 mg every 8 h
[8]	Randomized, open-label prospective trial	2011–2013	Febrile neutropenia in patients with acute leukemia or MDS-refractory anemia with excess blasts	65	68	57	56	Doripenem 1 g every 8 h	Meropenem 1.0 g every 8 h

MDS, myelodysplastic syndrome; UTI, urinary tract infection; RCT, randomized controlled trial.

## Appendix A: List of Terms of the Search Strategy

Pubmed

1. “doripenem” [MeSH Terms]

2. “doripenem” [All Fields]

3. 1 OR 2

4. “infection” [MeSH Terms]

5. “infection” [All Fields]

6. 4 OR 5

7. “randomized” [All Fields]

8. “randomised” [All Fields]

9. 7 OR 8

10. 3 AND 6 AND 9

Embase

1. “doripenem”/exp

2. “doripenem”

3. 1 OR 2

4. “infection”

5. “randomized”

6. “randomised”

7. 5 OR 6

8. 3 AND 4 AND 7

Cochrane

1. doripenem

2. infection

3. #1 AND #2

## References

[1] Kalil A.C., Metersky M.L., Klompas M., Muscedere J., Sweeney D.A., Palmer L.B., Napolitano L.M., O’Grady N.P., Bartlett J.G., Carratalà J. (2016). Management of adults with hospital-acquired and ventilator-associated pneumonia: 2016 Clinical practice guidelines by the Infectious Diseases Society of America and the American Thoracic Society. Clin. Infect. Dis..

[2] Mazzei T. (2010). The pharmacokinetics and pharmacodynamics of the carbapanemes: Focus on doripenem. J. Chemother..

[3] Castanheira M., Jones R.N., Livermore D.M. (2009). Antimicrobial activities of doripenem and other carbapenems against *Pseudomonas aeruginosa*, other nonfermentative bacilli, and *Aeromonas* spp.. Diagn. Microbiol. Infect. Dis..

[4] Douraghi M., Ghalavand Z., Nateghi Rostami M., Zeraati H., Aliramezani A., Rahbar M., Mohammadzadeh M., Ghourchian S., Boroumand M.A., Abdollahi A. (2016). Comparative *in vitro* activity of carbapenems against clinical isolates of *Acinetobacter baumannii*. J. Appl. Microbiol..

[5] Li Y., Lv Y., Xue F., Zheng B., Liu J., Zhang J. (2015). Antimicrobial resistance surveillance of doripenem in China. J. Antibiot. Tokyo.

[6] Qu X.Y., Hu T.T., Zhou W. (2015). A meta-analysis of efficacy and safety of doripenem for treating bacterial infections. Braz. J. Infect. Dis..

[7] Wagenlehner F.M., Sobel J.D., Newell P., Armstrong J., Huang X., Stone G.G., Yates K., Gasink L.B. (2016). Ceftazidime-avibactam versus doripenem for the treatment of complicated urinary tract infections, including acute pyelonephritis: RECAPTURE, a phase 3 randomized trial program. Clin. Infect. Dis..

[8] Oyake T., Takemasa-Fujisawa Y., Sugawara N., Mine T., Tsukushi Y., Hanamura I., Fujishima Y., Aoki Y., Kowata S., Ito S. (2019). Doripenem versus meropenem as first-line empiric therapy of febrile neutropenia in patients with acute leukemia: A prospective, randomized study. Ann. Hematol..

[9] Higgins J.P., Altman D.G., Gotzsche P.C., Juni P., Moher D., Oxman A.D., Savovic J., Schulz K.F., Weeks L., Sterne J.A. (2011). The Cochrane Collaboration’s tool for assessing risk of bias in randomised trials. BMJ.

[10] Rea-Neto A., Niederman M., Lobo S.M., Schroeder E., Lee M., Kaniga K., Ketter N., Prokocimer P., Friedland I. (2008). Efficacy and safety of doripenem versus piperacillin/tazobactam in nosocomial pneumonia: A randomized, open-label, multicenter study. Curr. Med. Res. Opin..

[11] Kollef M.H., Chastre J., Clavel M., Restrepo M.I., Michiels B., Kaniga K., Cirillo I., Kimko H., Redman R. (2012). A randomized trial of 7-day doripenem versus 10-day imipenem-cilastatin for ventilator-associated pneumonia. Crit. Care.

[12] Chastre J., Wunderink R., Prokocimer P., Lee M., Kaniga K., Friedland I. (2008). Efficacy and safety of intravenous infusion of doripenem versus imipenem in ventilator-associated pneumonia: A multicenter, randomized study. Crit. Care Med..

[13] Naber K.G., Llorens L., Kaniga K., Kotey P., Hedrich D., Redman R. (2009). Intravenous doripenem at 500 milligrams versus levofloxacin at 250 milligrams, with an option to switch to oral therapy, for treatment of complicated lower urinary tract infection and pyelonephritis. Antimicrob. Agents Chemother..

[14] Tazuma S., Igarashi Y., Inui K., Ohara H., Tsuyuguchi T., Ryozawa S. (2015). Clinical efficacy of intravenous doripenem in patients with acute biliary tract infection: A multicenter, randomized, controlled trial with imipenem/cilastatin as comparator. J. Gastroenterol..

[15] Lucasti C., Jasovich A., Umeh O., Jiang J., Kaniga K., Friedland I. (2008). Efficacy and tolerability of IV doripenem versus meropenem in adults with complicated intra-abdominal infection: A phase III, prospective, multicenter, randomized, double-blind, noninferiority study. Clin. Ther..

[16] Kollef M.H., Nathwani D., Merchant S., Gast C., Quintana A., Ketter N. (2010). Medical resource utilization among patients with ventilator-associated pneumonia: Pooled analysis of randomized studies of doripenem versus comparators. Crit. Care.

[17] Kongnakorn T., Mwamburi M., Merchant S., Akhras K., Caro J.J., Nathwani D. (2010). Economic evaluation of doripenem for the treatment of nosocomial pneumonia in the US: Discrete event simulation. Curr. Med. Res. Opin..

[18] Zilberberg M.D., Mody S.H., Chen J., Shorr A.F. (2010). Cost-effectiveness model of empiric doripenem compared with imipenem-cilastatin in ventilator-associated pneumonia. Surg. Infect. Larchmt.

[19] Drzewiecki A., Bulanda M., Talaga K., Sodo A., Adamski P. (2012). Comparison of *in vitro* activity of doripenem, imipenem and meropenem against clinical isolates of *Enterobacteriaceae, Pseudomonas* and *Acinetobacter* in Southern Poland. Pol. Przegl. Chir..

[20] Firsov A.A., Gilbert D., Greer K., Portnoy Y.A., Zinner S.H. (2012). Comparative pharmacodynamics and antimutant potentials of doripenem and imipenem with ciprofloxacin-resistant *Pseudomonas aeruginosa* in an *in vitro* model. Antimicrob. Agents Chemother..

[21] Wali N., Mirza I.A. (2016). Comparative In Vitro Efficacy of doripenem and imipenem against multi-drug resistant *Pseudomonas aeruginosa*. J. Coll. Physicians Surg. Pak..

[22] Mustafa M., Chan W.M., Lee C., Harijanto E., Loo C.M., Van Kinh N., Anh N.D., Garcia J. (2014). A PROspective study on the Usage patterns of Doripenem in the Asia-Pacific region (PROUD study). Int. J. Antimicrob. Agents.

[23] Chao C.M., Chen C.C., Huang H.L., Chuang Y.C., Lai C.C., Tang H.J. (2016). Clinical experience of patients receiving doripenem-containing regimens for the treatment of healthcare-associated infections. PLoS ONE.

[24] Luyt C.E., Aubry A., Lu Q., Micaelo M., Brechot N., Brossier F., Brisson H., Rouby J.J., Trouillet J.L., Combes A. (2014). Imipenem, meropenem, or doripenem to treat patients with *Pseudomonas aeruginosa* ventilator-associated pneumonia. Antimicrob. Agents Chemother..

[25] Muscedere J.G., Day A., Heyland D.K. (2010). Mortality, attributable mortality, and clinical events as end points for clinical trials of ventilator-associated pneumonia and hospital-acquired pneumonia. Clin. Infect. Dis..

